# Health Science Community Will Miss This Bright and Uniting Star: In Memory of Professor Gjumrakch Aliev, M.D, Ph.D.

**DOI:** 10.3390/cancers13081965

**Published:** 2021-04-19

**Authors:** Vladimir N. Chubarev, Narasimha M. Beeraka, Mikhail Y. Sinelnikov, Kirill V. Bulygin, Vladimir N. Nikolenko, Elizaveta Mihaylenko, Vadim V. Tarasov, Liudmila M. Mikhaleva, Palmiro Poltronieri, Vijaya Padma Viswanadha, Siva G. Somasundaram, Cecil E. Kirkland, Kuo Chen, Junqi Liu, Ruitai Fan, Mohammad Amjad Kamal, Alexander A. Mironov, SubbaRao V. Madhunapantula, Etheresia Pretorius, Sergey V. Dindyaev, Cristian Muresanu, Olga A. Sukocheva

**Affiliations:** 1 Faculty of Pharmacology, Sechenov First Moscow State Medical University (Sechenov University), St. Trubetskaya, 8, bld. 2, 119991 Moscow, Russia; tchoubarov@mail.ru (V.N.C.); bnmurthy24@gmail.com (N.M.B.); mikhail.y.sinelnikov@gmail.com (M.Y.S.); kirill-bh-red@yandex.ru (K.V.B.); vn.nikolenko@yandex.ru (V.N.N.); elizavetamikhaylenko@gmail.com (E.M.); tarasov-v-v@mail.ru (V.V.T.); 2 Center of Excellence in Molecular Biology and Regenerative Medicine (CEMR), Department of Biochemistry, JSS Medical College, JSS Academy of Higher Education and Research (JSS AHER), Bannimantapa, Sri Shivarathreeshwara Nagar, Mysuru, Karnataka 570 015, India; mvsstsubbarao@jssuni.edu.in; 3 Faculty of Medicine, M.V. Lomonosov Moscow State University, 117192 Moscow, Russia; 4 Research Institute of Human Morphology, 3 Tsyurupy Street, 117418 Moscow, Russia; mikhalevalm@yandex.ru; 5 Institute of Sciences of Food Productions, National Research Council of Italy, via Monteroni km 7, 73100 Lecce, Italy; palmiro.poltronieri@ispa.cnr.it; 6 Department of Biotechnology, Bharathiar University, Coimbatore, Tamil Nadu 641 046, India; padma.vijaya@gmail.com; 7 Department of Biological Sciences, Salem University, Salem, WV 26426, USA; siva.somasundaram@salemu.edu (S.G.S.); ekirkland@salemu.edu (C.E.K.); 8 Cancer Center, Department of Radiation Oncology, the First Affiliated Hospital of Zhengzhou University, Zhengzhou 450000, China; chenkchenk@foxmail.com (K.C.); jqliu2@yahoo.com (J.L.); fanruitai@126.com (R.F.); 9 West China School of Nursing/Institutes for Systems Genetics, The Frontier Science Center for Disease-Related Molecular Network, West China Hospital, Sichuan University, Chengdu 610041, Sichuan, China; prof.ma.kamal@gmail.com; 10 King Fahd Medical Research Center, King Abdulaziz University, P. O. Box 80216, Jeddah 21589, Saudi Arabia; 11 Enzymoics, 7 Peterlee Place, Novel Global Community Educational Foundation, Hebersham, NSW 2770, Australia; 12 Laboratory of Electron Microscopy, The FIRC Institute of Molecular Oncology, Via Adamello 16, 20139 Milan, Italy; mironovalexandre@gmail.com; 13 Department of Physiology, Faculty of Health Sciences, University of Pretoria, Arcadia 0007, South Africa; resiap@sun.ac.za; 14 Department of Histology, Embryology & Cytology, Pediatric Faculty, Federal State Budgetary Educational Institution of Higher Education “Ivanovo State Medical Academy” of the Ministry of Healthcare of the Russian Federation (FSBEI HE IvSMA MOH Russia), 8 Sheremetyevsky Ave., 153012 Ivanovo, Russia; dindyaev@mail.ru; 15 Research Center for Applied Biotechnology in Diagnosis and Molecular Therapies, Str. Trifoiului nr. 12 G, 400478 Cluj-Napoca, Romania; cristim23@gmail.com; 16 Discipline of Health Sciences, College of Nursing and Health Sciences, Flinders University of South Australia, Adelaide 5001, Australia

It is with deep sadness that we offer our memorial on the unexpected demise of our dear colleague, Professor Gjumrakch Aliev. He passed away in December 2020. He was 62.

Professor Aliev left us far too early. He had many projects underway that he looked forward to completing to better serve the world and all of its people. The scientific community has lost a bright, multidisciplinary scientist who could connect people with similar research interests across borders and continents.

Gjumrakch (Figure 1) was the internationally recognized founder of Gally International Research Institute (http://gallyinternational.com/about/gjumrakch, accessed on 20 February 2021), a professor at the Department of Anatomy of Sechenov University (https://www.sechenov.net/department-of-human-anatomy/, accessed on 20 February 2021), chairman of the International Division of the EuroEspes Biomedical Research Center (La Coruna, Spain), leading researcher (Full Professorship) at the Institute of Physiologically Active Compounds (http://www.istc.int/en/institute/8711, accessed on 20 February 2021), “Bentham Brand Ambassador” for the Russian Federation in the United Nations Organization (UNESCAP) and the committee on Science, Technology and Innovation (STI) for UNESCAP.

Professor Aliev was born on 1 September 1958 in Azerbaijan, of the former USSR. He graduated from high school with Gold Medal in 1976 (Nakhichevan, USSR). In 1982, he graduated summa cum laude from the Azerbaijan Medical Institute in Baku, Azerbaijan, receiving an M.D. in general medicine and health sciences (Honor Diploma). As a student, he was interested in research work and presented at conferences. He received First Award at International and USSR Medical Students Conference and Congress (1978–1979, Moscow, 1980 Kaunas, Latvian Republic; 1981 Saratov Russia, and 1982 Sank-Petersburg, Russia). In 1989, he received his PhD summa cum laude in Cardiovascular Biology and Pathology from the Ivanovo Medical Institute while conducting his research at the Moscow State University and the Russian Cardiology Research Center. Following this, Gjumrakch received postdoctoral training under the prestigious British Heart Foundation Grant Program in the University College London (advisor Professor Geoffrey Burnstock), specializing in medicine.

Professor Aliev possessed an encyclopedic knowledge in a wide range of scientific fields; however, his main research focus was linked to the development of unique technologies and treatment protocols for age-associated diseases. He was recognized internationally for his work in the fields of gerontology [1,2,3,4,5], oncology [6,7,8,9,10,11,12,13,14,15], cardiovascular diseases [16,17,18,19], endocrinology [2,20,21], and neurodegenerative diseases [15,20,22,23,24,25,26,27,28,29,30,31,32]. His research publications on the role of vascular and mitochondrial factors in the pathogenesis of aging [2,3,5], atherosclerosis [33,34], ischemia-reperfusion [18,35,36,37], stroke [4,18,35,38], and Alzheimer’s disease (AD) [23,24,33,39,40,41,42,43] are often cited (Table 1). Additionally, Professor Aliev and his colleagues were the first to propose the role of the energy crisis as a driving force for the acceleration of aging [6,35,44]. He authored and co-authored more than 400 peer-reviewed journal articles and book chapters (https://www.researchgate.net/profile/Gjumrakch_Aliev/research (accessed on 20 February 2021)), as well as over 170 scientific abstracts of conference presentations on neurodegenerative disease research, cardio- and cerebrovascular diseases, cancer, and electron microscopy. He also had several patents and rationalizations. Prior to his passing, Professor Aliev was the Project Director and Lead Investigator of several international scientific projects.

The idea of drug optimization in the treatment of several pathological conditions associated with aging and inflammation was the core aim of many of Prof. Aliev’s research projects. Elderly patients in all countries are prescribed a handful of drugs that often initiate side effects and provoke further prescription of additional supplements. Polypharmacy, a habit and/or a necessity to take too many drugs, is accompanied by a financial burden and health risks. Considering the advantage of less harmful and proven-to-be-beneficial chemicals, Prof. Aliev’s work strongly supports the development of oxidative stress-targeting and multi-purpose therapies as an approach to cure age-associated pathologies.

Professor Aliev had superior expertise in various aspects of microscopic analysis. Having had many years of experience in research and teaching with light microscopy, electron microscopy (EM), 2-photon microscopy, atomic force, and confocal microscopy, he produced pioneering work in different areas of EM-based techniques. These techniques included cytological in situ hybridization at the light and electron microscopic levels using non-isotopic colloidal gold probes [35,44], peroxidase-anti-peroxidase [44,45,46], and pre- and post-embedding single, double, and triple immunogold cytochemistry and quantification [37,39]. Professor Aliev’s Gally International Research Institute provided high quality scientific expertise in cellular, subcellular, functional, and biochemical assessments.

Professor Aliev enthusiastically engaged in collaborations, which led to him establishing connections among scientists all over the world. Through his cooperative and inclusive style of work, he was able to gather and link scientists with common research interests and complementary expertise from countries as diverse as China, India, USA, Russia, Singapore, Australia, and Germany. For instance, collaboration with Prof. SubbaRao V. Madhunapantula (Center of Excellence in Molecular Biology and Regenerative Medicine (CEMR Laboratory), JSS Medical College, JSS Academy of Higher Education & Research, Karnataka, India) resulted in a successful publication of several review articles in high-impact journals, including Frontiers in Immunology [47], Seminars in Cancer Biology [48], and International Journal of Molecular Sciences [49]. In just a year, his collaboration with Prof. Madhunapantula produced a strong research breakthrough and lead to the submission of multi-centric grant proposals. For all co-authors, it was a pleasure to work with Prof. Aliev as all his colleagues could feel through email letters his warm care and attention to all their needs. We will miss his timely actions and quick responses to all e-mail communications. Was he online 24/7? It certainly looked like he was. His early death is a huge loss to the scientific community.

Professor Aliev possessed prodigious administrative skills and had excellent rapport with his colleagues, friends, employers, and subordinates. Many scientific journals will be missing his continuing contributions. Professor Aliev served as an editor and editorial board member for many prestigious journals and as a grant review board member and reviewer for international granting agencies and foundations. He was the editor-in-chief of such journals as Central Nervous System Agents in Medicinal Chemistry, Applied Cell Biology, World Journal of Neuroscience, Open Journal of Psychiatry, Journal of Aging Science, Cardiovascular and Hematological Agents in Medicinal Chemistry, and Immunology, Endocrine and Metabolic Agents in Medicinal Chemistry. His work was cited about 25,000 times by nearly 10,000 documents (*h*-index 53; https://scholar.google.com/citations?user=a_TYBosAAAAJ&hl=en (accessed on 20 February 2021)) and he was among the top 2% of the world’s most-cited academic authors in the medical and health sciences (Stanford University database 2020; https://data.mendeley.com/datasets/btchxktzyw/2 (accessed on 20 February 2021)).

Professor Aliev was an active, honorable, contributing member of numerous scientific societies including American Association for the Advancement of Science (Neuroscience), International Society of Pharmacogenomics, American Association of Neuropathologist, Alzheimer Research Forum, Royal Society of Medicine (England), World Association of Neurotechnology, Science Advisory Board for Microscopy Society of Northeastern Ohio, Inc., USA, Italian Society of Electron Microscopy, Microscopy Society of America (New York Academy of Sciences), Russian Society of Microvascular Research, Russian Regional Society for Study of Atherosclerosis and Peripheral Vascular Diseases, Russian Society of Electron Microscopy, Russian Society of Atherosclerosis Investigation, and Russian Society of Anatomy, Histology and Embryology (Figure 2).

Despite the great demands of his life’s work, Professor Aliev always found time for his family. He cared greatly for his parents, visiting his mother in Ivanovo as often as possible while she was in declining health. He was a kind and loving presence in the life of his daughter Galina and his grandson Daniel. Daniel had a special connection to his grandfather and called him the “best grandfather in the world”. Prof. Aliev’s only daughter, Galina Alieva, wrote: “My father placed a great emphasis on the importance of education in my life for which I am eternally thankful. He supported my passion for the study of foreign languages which has, in turn, allowed me to become a professional in my job today. I had a very trustworthy and caring relationship with my father throughout my life. He is greatly missed by both myself and my son Daniel, his favourite and only grandson”. Gjumrakch continued to help his relatives who remained in Azerbaijan. He will be missed not only by his family and friends, but also by his large circle of colleagues and former students.

Professor Aliev was a remarkable teacher and an invaluable resource for medical students. He was involved in the teaching and development of numerous teaching materials and courses, as shown in Table 2. He developed research and educational programs in neuroscience, neurodegeneration, mitochondrial research, cardiology, cerebrovascular pathology, anatomy, histology, cancer, electron microscopy, and others. His enthusiasm for scientific progress extended to helping young researchers get their work published in high impact factor journals, a major hurdle for non-English-speaking scientists. He was keen to nurture students in all countries and all universities where he taught. He also managed to help with the promotion of the careers of young talented scientists. Gjumrakch’s devotedly helped to prosper young researchers who struggled to publish in English. Many of Professor Aliev’s pupils have gone on to successful scientific careers and continue the development of his scientific ideas. Among his graduate students were Dr. E. Bedyaev, Dr. A. Mironov Jr., Dr. S. Gurkin, Mr. K. Arun Raina, Dr. Mark A. Obrenovich, Mr. Justin Shenk, and Mr. Gerardo Pacheco, Ms. Celia Cobb, Mr. Hector Palacios, Mrs. Brianna Walrafen, Ms. Amanda Lipsitt, and Mr. Andres Aguirre. Professor Aliev also supervised postdoctoral scientists and was involved in clinical training of recent medical graduates (2003–2020), including Dr. Dilara Seyidova, Dr. Mariana Rosca, Dr. Richard F. Silver, Dr. Ali Aliyev, Dr. Nizami Rzayev, and Dr. Andra Mardale. We sincerely apologize if we missed anybody.

Professor Aliev received numerous awards and honorable recognitions for his work, including the prestigious Upjohn Scientific Prize Award from Italian Pharmacological Society (1994; Torino, Italy), Outstanding Scholars Award/the 20th Century Honor Diploma Cambridge (1999, UK), Honorary Research Board award of Advisory of The American Biographical Institute (1999), George W. Bush Foundation Fellowship (2002), Outstanding Leadership Honor Diploma of American Biographical Institute (2003), Commemorative Medal Man of the Year 2004 American Biographical Institute, UTSA Student Organization Consul Recognition Honor Advisor Diploma (Journal of the College of Science, UTSA; 2008), Pontificia Universidad Javeriana, Facultad Ciencias Honor Diploma (Lectures Series: Theory and Practice of Modern Electron Microscopy Application for Biology and Medicine; 2008, Bogota, Colombia), and OMICS Group Special Honor diploma in Appreciation of Esteemed Editorial Support (2014, San Antonio, TX, USA).

The most recent scientific interests of Professor Aliev were in the fields of cancer cell biology, biochemistry, and the functional morphology of cells and tissues. He investigated the structure and functions of endothelial cells [33,35,44,50], smooth muscle cells [51,52], neurons [3,45,53,54], glial cells [55,56,57,58], and macrophages [47,50]. He was keen to address the most urgent health problems. Last year (2020), he generated ideas for how to target SARS-CoV-2 associated complications [47]. However, the main focus of his research was set on deciphering the mechanisms of atherogenesis, ischemia/reperfusion, tumor angiogenesis, signal transduction, mitochondrial DNA deletion, cancer growth, and metastasis [37,48,59,60,61,62,63]. His laboratory used transgenic mouse models in vivo and a large variety of cutting-edge in vitro molecular biology methods [32,35,44,46]. During the last few years, Professor Aliev was investigating the interaction of nanoparticles with tissues and cells [11,16,49,64,65]. His work aimed to elucidate the pathogenic mechanisms underlying nanoparticles’ effects and to discover potential new drug development strategies. For instance, his in vitro cancer cell model for the peptide based new drug development study showed promising results regarding the specific delivery of drugs to tumor tissues [63,65]. Another of his in vivo studies found that nanoparticles are able to cross the blood–brain barrier, which has been the biggest impediment in delivering drugs to patients with Alzheimer’s disease [16,18,64].

Prof. Aliev was named as the Primary Investigator and Co-Investigator on numerous grant applications and his research projects were supported financially as indicated by successful grant rewards in recent years. His recent projects were supported by the Russian Academy of Sciences (RSCF No. 14-23-00160P, 2 016–2020; Institute of Physiological Active Compounds, Russia), Stress Relief and Memory Center fund (New York, USA; “Stress relief and memory training in conjunction with selective natural antioxidants as an alternate method for treatment of age associated mental retardation, depression, and cancer” 2016–2019), Ministry of Science, Technology, and Innovation of Russian Federation with International Cooperation Foundation (“Unified Technology for the Evaluation of the Effectiveness of the supramolecular conjugates for the inhibition of the reverse cellular transport in Cancer and CNS Diseases”, 2018–2020), Skolkovo Foundation (Russian Skolkovo Innovation Center; 2018–2020), Nine Sigma (Japan; Project Code: 923392; 2018–2020), and Brain Tumor Foundation (USA, “Evaluation of Mitochondrial DNA Overproliferation and Deletion as an Early Diagnostic Marker and Therapeutic Target for the Brain Tumor”, 2018–2020). Unfortunately, Prof. Aliev’s company, Gally International, will continue to run his clinical projects without him. The above overview provides only a brief synopsis of his remarkable achievements.

In conclusion, we regret that this article cannot reflect in full the bright personality and dynamic force that Grjumrakch possessed. Gjumrakch cared deeply about his associates. He was always ready to help with careers, research studies, and publications, as well as with health and fitness. He will be remembered by his colleagues and students as a remarkable scientist and enthusiastic educator, whose students will continue the development of his ideas at universities and research centres in India, China, Russia, USA, and Europe.

**Table 1:** Top 10 Prof. Aliev’s highly cited articles in the field of Alzheimer’s disease and neuroinflammation. Source of information; https://scholar.google.com/citations?user=a_TYBosAAAAJ&hl=en (accessed on 20 March 2021).

Title, Reference	# of Times Cited on 20 March 2021	Year of Publication
Oxidative damage is the earliest event in Alzheimer disease [22]	1961	2001
Mitochondrial abnormalities in Alzheimer’s disease [39]	1375	
Activation and redistribution of c-jun N-terminal kinase/stress activated protein kinase in degenerating neurons in Alzheimer’s disease [45]	449	
Role of mitochondrial dysfunction in Alzheimer’s disease [23]	379	2002
Is oxidative damage the fundamental pathogenic mechanism of Alzheimer’s and other neurodegenerative diseases? [24]	358	
The role of oxidative stress in the pathophysiology of cerebrovascular lesions in Alzheimer’s disease [44]	205	
Microtubule reduction in Alzheimer’s disease and aging is independent of τ filament formation [66]	327	2003
Vascular oxidative stress in Alzheimer disease [67]	209	2007
Nucleic acid oxidation in Alzheimer disease [41]	210	2008
Oxidative stress mediated mitochondrial and vascular lesions as markers in the pathogenesis of Alzheimer disease [33]	148	2014
>Guidelines for the use and interpretation of assays for monitoring autophagy [68]	9455	2021

**Table 2:** Aliev’s Teaching Activities during 1986–2015.

Institution	Years	Subject, Course Title
Ivanovo Medical Institute, Russia	1986–1990	Cytology, Histology, and Embryology (Microanatomy) (Biology and Medical courses)
	1988–1990	Anatomy (Biology and Medical courses)
	1989–1990	Neuroscience (Biology and Medical courses)
University of Jaen, Spain	1996–1997	Cytology and Histology (Biology course)
Case Western Reserve University (CWRU) Cleveland, OH, USA	1998–1999	Cytology and Histology (Biology course)
	1998–1999	Vascular Biology (Biology and Medical courses)
	1998–1999	Neuroscience (Biology and Medical Students)
	1999–2003	Clinical Biochemistry: Molecular Mechanisms of Cardiovascular and Neurodegenerative Disease Pathogenesis
	2004–2005	General and Systemic Pathology for Medical students
	2004–2006	Application of Electron Microscopy for Biology and Medicine (Biology and Medical courses)
University of Texas at San Antonio, San Antonio, TX, USA	2006–2009	Application of Electron Microscopy for Biology and Medicine (Biology and Biotechnology courses)
	2008–2009	Cytoskeleton and Disease (undergraduate and graduate courses)
	2008–2009	Aging and the Nervous System (undergraduate and graduate students)
Pontificia Universidad Javeriana, Bogotá, Colombia	2009–2010	Biochemistry of Aging and Aged Associated Diseases
	2009–2010	Clinical Advanced Biochemistry of Cardiovascular system and CNS
	2009–2010	Application of Electron Microscopy in Biology and Medicine
University of Atlanta, Atlanta, GA, USA	2010–2015	Health Sciences and Healthcare Administration: HC605; HS610; HS615 (BSc and MS students)

## Figures and Tables

**Figure 1:**
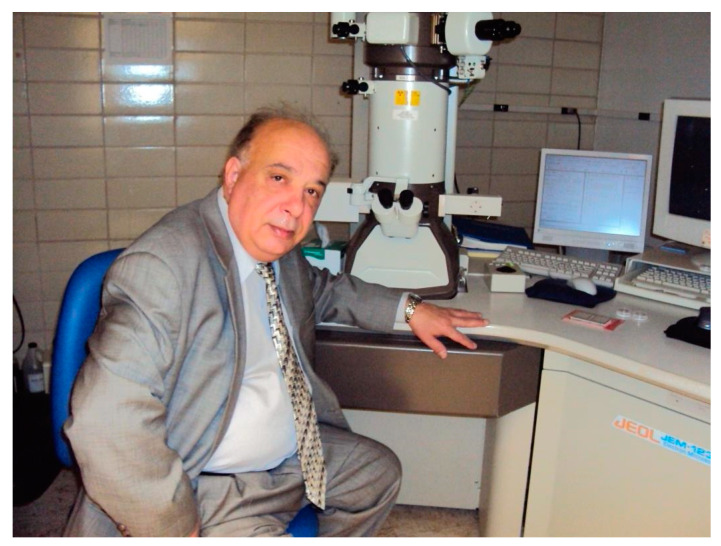
Professor Aliev in his Laboratory, Sechenov University, Moscow (2019).

**Figure 2:**
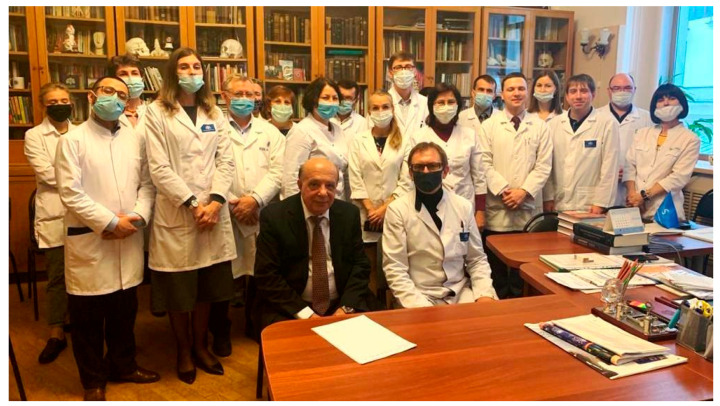
Visiting Professor Aliev, Professor Nikolenko (sitting at the table in front), and staff members of Department of Human Anatomy at Sechenov Universityff, Moscow, 2020. From left to right in the first row (front): Genrik G. Verdiyan, Mariya A. Zolotareva, Ivan N. Chairkin, Olga N. Kovaleva, Ekaterina S. Klyukina, Nurgozel K. Akyeva, Yuri O. Zharikov, Kirill V. Bulygin, Karina A. Vasyanina; In the second row (behind): Anna V. Olsufieva, Angela D. Vovkogon, Inna V. Merenkova, Natalia V. Chairkina, Ivan V. Shevchuk, Andrey V. Suslov, Ruslan Z. Nurimanov, Sania N. Odinokova, Alexey E. Strizhkov.
